# An Unusually Large Oral Lipoma of Buccal Mucosa and Vestibulum: A Case Report

**DOI:** 10.1155/crid/3913290

**Published:** 2026-02-10

**Authors:** Petra Stazić, Mislav Ušljebrka, Ante Mihovilović, Ante Pojatina, Andrija Radoš, Jure Martinić, Daniel Jerković

**Affiliations:** ^1^ Department of Maxillofacial Surgery, University Hospital of Split, Split, Croatia; ^2^ Study of Dental Medicine, School of Medicine, University of Split, Split, Croatia, unist.hr

**Keywords:** case report, excision biopsy, lipoma, oral pathology, oral surgery

## Abstract

Lipoma is a benign tumor of fatty tissue. Although lipomas are common tumors of mesenchymal origin in the human body, they rarely occur in the oral and maxillofacial region with a prevalence of 1%–4%. Even though lipomas in the oral cavity can appear anywhere, their etiology remains unclear. These tumors grow slowly and are painless, soft, and often encapsulated. Nevertheless, this tumor, with its growth, can cause speech, chewing, and denture fabrication difficulties and can be associated with pain, swelling, and numbness. Since it can be differentially diagnosed with liposarcoma, the preferred treatment for this tumor is adequate surgical excision, which is associated with a low recurrence rate. This report presents the clinical case of a large oral lipoma located in buccal mucosa and vestibule of the oral cavity that has been growing for the past 8 years. Surgical excision of the lipoma was performed under local anesthesia, requiring precise dissection due to its proximity to the mental nerve to minimize potential nerve damage. At the 3‐month follow‐up examination, the clinical findings were normal, with no signs of recurrence and no paresthesia in the operated area.

## 1. Introduction

A lipoma is a benign tumor usually composed of mature adipocytes that develops from mesenchymal connective tissue [1]. Although lipomas are the most common mesenchymal neoplasms, only around 17% of soft tissue lipomas are in the oral and maxillofacial region [2]. The oral cavity is one of the rarest sites for lipomas with an overall incidence of 1%–4% [3–5]. Oral lipomas can appear in different anatomical locations, mostly in buccal mucosa, followed by tongue, palate, lip, floor of the mouth, vestibulum, retromolar area, and gingiva [5, 6]. They are more common in the age group over 50 years, with no significant difference in prevalence between the sexes [3, 7, 8]. Histologically, they can be classified as simple lipomas, fibrolipomas, angiolipomas, spindle cell lipomas, pleomorphic lipomas, intramuscular or infiltrating lipomas, chondrolipomas, salivary gland lipomas, myxoid lipomas, and atypical lipomas [1, 9]. Oral lipomas usually present as benign, slow‐growing, soft and smooth‐surfaced nodular masses [1, 8, 10]. The average size of lipomas found in the oral cavity is approximately 2 cm [3, 7, 8]. Although small lipomas in the intraoral cavity are mostly asymptomatic, large tumors may cause symptoms depending on their location, development, and growth rate resulting in discomfort, difficulty speaking and mastication, or disfigurement [11]. Here, we report a case of large intraoral lipoma on buccal mucosa and vestibulum enveloping the mental nerve.

## 2. Case Report

**Figure 1:**
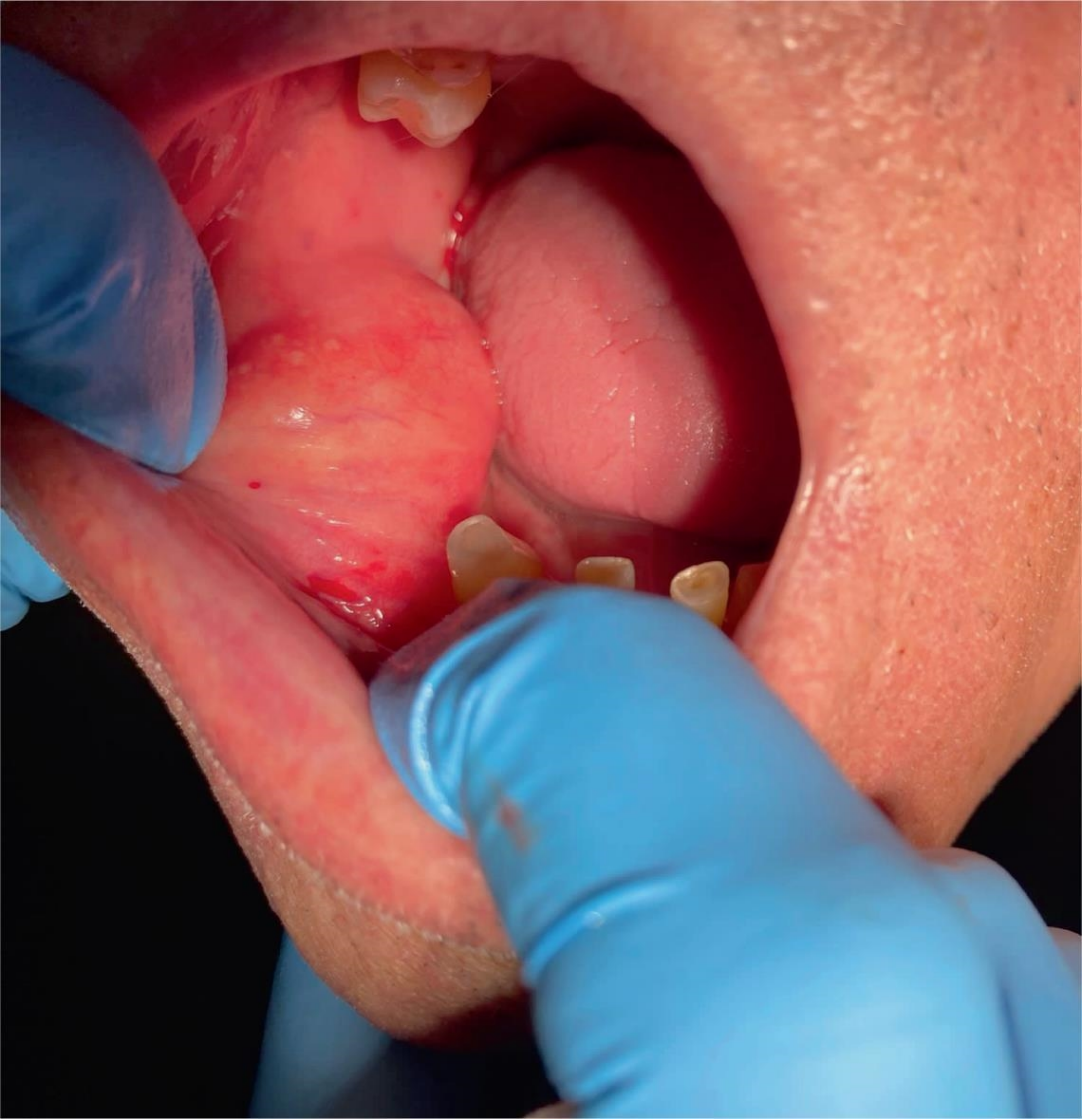
Intraoral view of lobulated, soft fluctuant swelling mass in the right buccal mucosa and vestibulum.

A 73‐year‐old male patient came to the Department of Oral Surgery at the University Hospital of Split, in Split, Croatia, for a specialist examination due to an intraoral mass in the region of the right buccal mucosa and vestibulum. The patient was referred to an oral surgeon for an examination by his chosen dentist due to a lesion that was presenting a disturbance for making a new dental prosthesis. A thorough medical history was taken, during which the patient denied any systemic diseases, as well as any history of trauma in his oral cavity. The patient did not have any symptoms or complaints related to the oral changes. Additionally, the patient has noticed that the lesion has been growing slowly and has been present for 8 years. Extraoral examination was normal, without pain and swollen cervical lymph nodes. Intraoral examination revealed a large oval mobile mass in the edentulous region in the right buccal mucosa and buccal vestibulum which was not painful. On palpation, the nature of the swelling was a fluctuant, lobulated mass and nontender (Figure 1). The oral mucosa over the mass was normal in texture without ulceration or inflammation. Fine needle aspiration biopsy showed negative findings.

**Figure 2:**
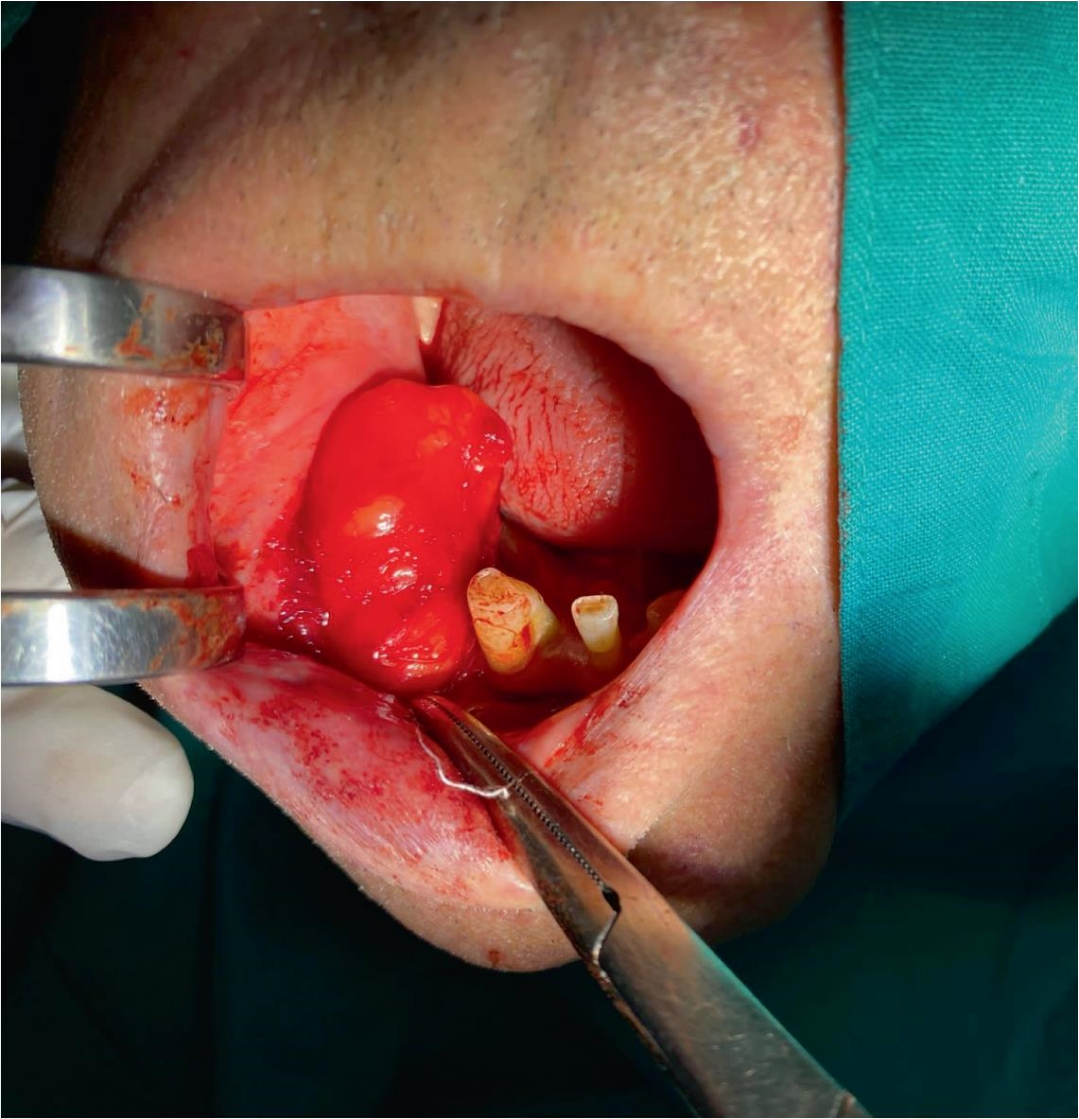
Intraoral view of the surgical removal of the entire mass by excisional biopsy.

**Figure 3:**
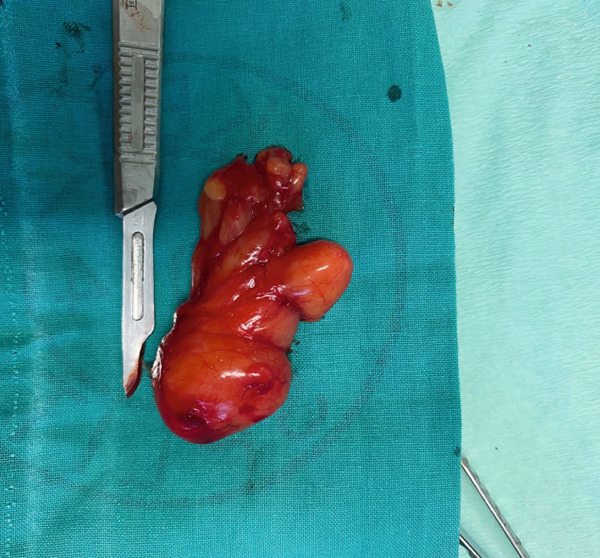
Excised, encapsulated yellowish mass.

**Figure 4:**
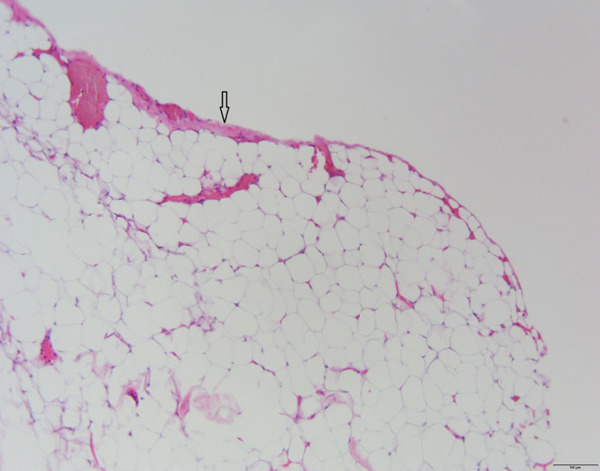
Proliferation of uniform, mature adipocytes with a thin fibrous capsule on the surface (arrow).

A surgical treatment was performed by an oral surgeon under local infiltration anesthesia with two cartridges of lidocaine–epinephrine (20 + 0.0125 mg/mL, Belupo, Croatia). The excision biopsy was made over the tumor mass on the right buccal mucosa. This revealed an oval yellowish tumor that was in close contact with the mental nerve with part of the tumor mass enveloping mental nerve and was carefully dissected from the nerve and surrounding tissue (Figure 2). The biopsy procedure resulted in the complete removal of the entire mass. Figure 3 shows the excised mass. After extirpation, the wound was closed using resorbable sutures 4‐0 (Coated VICRYL Plus Antibacterial [polyglactin 910], Ethicon US LLC) and a rubber drain was placed in the excision site. After the surgery, the patient was prescribed with 1 g of amoxicillin with clavulanic acid (875 + 125 mg, Klavocin bid, Pliva, Croatia) two times a day for 7 days. The excised mass was placed in 10% neutral buffered formalin and sent for histopathological examination. The specimen was measured 6 × 3 × 1.5 cm by the pathologist. Histologically, it consisted of mature adipocytes and its surface was covered by a capsule. The pathological diagnosis confirmed the diagnosis of a simple lipoma (Figure 4). At the follow‐up examination after 2 weeks, the wound has healed properly without any signs of infection or inflammation and then the rubber drain and sutures were removed; however, the patient developed a mild case of paresthesia of the right part of the chin and lower lip. The patient was therefore prescribed with a high dose of vitamin B complex (Neurobion Forte, P&G Health Austria GmbH & Co. OG, Spittal an der Drau, Austria). At the 3‐month follow‐up examination, the clinical findings were normal, with no signs of recurrence present and the numbness sensation was no longer present.

## 3. Discussion

Lipomas are the most common benign neoplasms composed of mature adipocytes, but they are relatively rare in the oral region [3]. In our case, the intraoral lipoma affected both buccal mucosa and the area of the buccal vestibulum. Buccal mucosa is shown to be the most common intraoral site where this tumor appears [7, 12]. It is assumed that uncontrolled growth of adipose tissue may play a role in the development of lipoma, and it could be related to the high amount of fatty tissue present in buccal mucosa [13, 14]. Still, the development of the lipoma in areas with low adipose tissue, such as the buccal vestibule, is explained by the possibility that fatty tissue may be formed through metaplastic transformation of connective tissue [15]. Although the etiology and pathogenesis of oral lipomas remain unclear, some studies associate lipomas with mechanical trauma, inflammation, obesity, radiation, and mucosal infections with chronic irritation acting as a possible trigger for its development [16, 17]. However, in our case, due to the size of the tumor and its asymptomatic nature, it is not clear which was the initial site of the tumor development, nor can it be associated with any etiological factor.

Superficial lipomas in the oral region, as in our case, can be easily diagnosed clinically since palpation reveals a soft, painless, and mobile mass. Deep lipomas usually are not palpable, and it can be hard to distinguish them from salivary gland tumors or other benign mesenchymal neoplasms [17, 18]. The clinical progression of oral lipomas is typically slow, painless, and gradual, with diagnosis often happening incidentally when the tumor size interferes with oral function or causes a cosmetic concern [3, 6]. This was also presented in our case when the size of the lesion, although asymptomatic, interfered with the making of a new dental prosthesis. Usually, the mean diameter of the oral lipomas at the time of diagnosis is approximately 2 cm, while bigger tumors are less frequent [3, 19]. However, the measurements of the lipoma in our case were 6 × 3 × 1.5 cm. Although this size of lipomas in the oral cavity is rare, these dimensions are not so strange considering its location in the buccal region along with longer progression time due to its asymptomatic nature [7, 8, 20].

The anamnesis of a tumor that has been growing for 8 years in our case also contributes to the fact that it presents most likely a less aggressive, benign type of tumor. However, despite its benign nature, due to its size and placement, removal of the tumor can cause damage of important structures if it is located near them [16]. In this presented case, the tumor circumscribed the mental nerve which was revealed during surgical removal although it was completely asymptomatic. In such cases, sensory deficits and neuralgic symptoms are possible complications of surgical removal [16, 21], as in our case, where a mild case of transitory paresthesia developed despite the careful separation during the excision. Also, removal of large lipomas in the oral and maxillofacial region can cause esthetic complications, and intraoral excision approach is preferred treatment providing no external scarring [22]. However, the most appropriate treatment for this tumor is complete surgical excision to prevent local recurrence. Although overall local recurrence of oral lipoma is 1%–2% [23], the infiltrating histopathological subtype, intramuscular lipoma, is more prone to recurrence demanding a wider excision [24]. Moreover, malignant lesions, such as oral liposarcoma, can easily be misdiagnosed as lipomas, which is important to keep in mind and requires excision without leaving a safety margin [25]. Also, the cheek is one of the most common oral locations of extranodal lymphomas which should be kept in mind when encountering asymptomatic swelling of the buccal mucosa [26]. More often, benign intraoral lipoma can be mistaken for a cyst or salivary gland tumor or even a dental infection [14, 27]. For the same reasons, although lipomas in the oral and maxillofacial region can usually be recognized clinically, histopathology continues to serve as the gold standard for a definitive diagnosis and to rule out malignancy. However, in our case, the histopathological analysis revealed a classic lipoma, which is the most common variant of intraoral lipomas, accounting for between 45% and 50% of all intraoral lipomas [28].

The patient in our case, although previously asymptomatic, was satisfied with the outcome of surgical treatment since it enabled the making of a dental prosthesis and restored him masticatory and esthetic function.

## 4. Conclusion

In summary, it can be concluded that oral lipoma is a rare neoplasm in the oral cavity. Its asymptomatic nature can lead to unnoticed growth and disproportionate size. The tumor growth is slow and painless, so patients typically come for examination when the tumor causes aesthetic disturbances and discomfort. For diagnosing this tumor, a detailed medical history and clinical examination are often sufficient. After an accurate diagnosis is made, it is followed by adequate surgical excision without a definite margin and histopathological examination to establish the final diagnosis, determine the histological type of oral lipoma, and rule out other pathology such as malignant liposarcoma.

## Author Contributions

P.S. participated in conceptualization, data curation, data interpretation, visualization, and writing the manuscript. M.U. participated in conceptualization, data curation, data interpretation, and writing the manuscript. A.M. participated in conceptualization, data curation, and drafting the manuscript. A.P. participated in conceptualization, data curation, data interpretation, and drafting the manuscript. A.R. participated in conceptualization, data interpretation, visualization, and writing the manuscript. J.M. participated in conceptualization, data interpretation, and writing the manuscript. D.J. participated in conceptualization, data interpretation, coordination, writing the manuscript, and supervision of the whole research.

## Funding

No funding was received for this research.

## Ethics Statement

No written consent has been obtained from the patients as there is no patient identifiable data included in this case report/series. This study has followed the Declaration of Helsinki on medical protocol and ethics.

## Conflicts of Interest

The authors declare no conflicts of interest.
